# A Study on the Battery Recycling Process and Risk Estimation

**DOI:** 10.3390/ijerph21121649

**Published:** 2024-12-10

**Authors:** Taeho Kim, Cheolhee Yoon, Seungho Jung

**Affiliations:** Department of Environmental and Safety Engineering, Ajou University, Suwon 16499, Republic of Korea; xogh7887@ajou.ac.kr (T.K.); cjfgml0509@ajou.ac.kr (C.Y.)

**Keywords:** battery, battery recycling, risk assessment, RAC matrix, hydrometallurgical process

## Abstract

The demand for the use of secondary batteries is increasing rapidly worldwide in order to solve global warming and achieve carbon neutrality. Major minerals used to produce cathode materials, which are key raw materials for secondary batteries, are treated as conflict minerals due to their limited reserves, and accordingly, research on the battery recycling industry is urgent for the sustainable secondary battery industry. There is a significant risk of accidents because there is a lack of prior research data on the battery recycling process and various chemicals are used in the entire recycling process. Therefore, for the safety management of related industries, it is necessary to clearly grasp the battery recycling process and to estimate the risk accordingly. In this study, the process was generalized using the information on the battery recycling process suggested in the preceding literature. And to estimate the relative risk of each battery recycling process, the RAC (Risk Assessment Code) matrix described in the US Department of Defense’s “MIL-STD-882E” was used. Severity was derived by using “NFPA 704”, and probability was derived by combining generalized event analysis for each process and the WEEE (Waste Electrical and Electronic Equipment) report. The results confirmed that the process using H_2_SO_4_ had the highest risk when extracting Li during the leaching process, and that dismantling and heat treatment had the lowest risk. Using the probability factor for each process calculated through the research, it is expected to be used in future battery recycling process research as basic data for quantitative risk assessment of the battery recycling process.

## 1. Introduction

Globally, the expansion of electric vehicle (EV) adoption is being promoted as part of policies to combat climate change, which has increased attention on the use of secondary batteries [1]. Life Cycle Assessment (LCA) studies comparing carbon emissions between internal combustion engine vehicles and electric vehicles show that the longer a vehicle is operated, the more advantageous it becomes in reducing carbon emissions through the use of secondary batteries [2]. Consequently, the market for electric vehicles, which utilize eco-friendly energy sources, is expanding in the automotive sector that traditionally relies on fossil fuels. By 2020, the global number of electric vehicles in circulation reached 11 million, a 100-fold increase compared to 2015 [3].

Most batteries used in electric vehicles are lithium-ion batteries (LIBs). Due to their high energy and power density, LIBs are widely used in electric vehicles, mobile phones and IT devices, energy storage systems (ESSs), and power tools [4]. LIBs offer advantages such as high voltage, high energy density, a low self-discharge rate, and a wide operating temperature range. However, they also pose safety risks, including overheating and fire hazards. Additionally, LIBs require long charging times and contain toxic chemicals that must be properly disposed of during battery disposal [5].

LIBs contain toxic organic electrolytes, including nickel and cobalt, which are classified as carcinogenic and mutagenic substances, posing environmental and health hazards. Despite these concerns, lithium and cobalt, which are used in the synthesis of cathode materials for LIBs, are limited resources [6]. The primary reason for recycling LIBs is the scarcity of lithium. Considering the need for efficient use of battery raw materials and environmental sustainability, it is crucial to properly process and recycle LIBs. Lithium is the most widely used metal in battery production, but its global reserves are limited. In South Korea, there are no domestic lithium resources, so the country must rely entirely on imports for battery production. However, as the battery market continues to grow, demand for lithium is outpacing supply, making it increasingly difficult to meet demand through imports alone. Therefore, recycling LIBs to recover lithium and other essential metals for battery production is important for preventing environmental pollution and ensuring stable raw material supply [7].

The global LIB recycling market is expected to reach USD 23.72 billion by 2030 [8]. As the battery recycling market grows, the frequency of accidents during the recycling process is also increasing. Notable incidents include a fire and explosion caused by waste aluminum foil in Ningxiang, Hunan Province, China, in January 2021, which resulted in one death and 20 injuries, and a major fire at the Fenix battery recycling plant in Kilwinning, Scotland, in 2023. These incidents highlight the frequent occurrence of accidents during the battery recycling process. Therefore, to improve the safety of battery recycling processes in line with the expanding market, it is necessary to conduct risk assessment studies based on the generalization of recycling processes through the analysis of previous research.

The battery recycling process is generally divided into pretreatment and post-treatment stages. The pretreatment process involves removing the risk of explosion and crushing the batteries, while the post-treatment process involves recovering metals from the crushed batteries. In the pretreatment stage, wet or dry discharging is carried out to deactivate the batteries, and in the post-treatment stage, hydrometallurgical or pyrometallurgical processes are used to recover metals [9]. Hydrometallurgy is a process that uses acidic or alkaline solutions to extract, refine, and recover metals. Since various chemicals are used in the hydrometallurgical process, there is a risk of chemical exposure.

Therefore, this study aims to conduct a risk assessment of the LIB recycling process by generalizing various battery recycling processes through analysis

## 2. Research Methodology

### 2.1. Analysis of Prior Research on Standardizing the Recycling Process

To standardize the battery recycling process, this study analyzed previous studies. The analyzed research papers are summarized in Table 1 below.

Through the analysis of these prior studies, a generalized schematic of the battery recycling process was derived for use in this study. Based on this generalized recycling process, a “risk estimation study” was conducted to identify the most hazardous processes. The risk estimation employed a matrix technique that combines severity and probability. Severity was calculated based on the NFPA fire diamond, and probability was estimated by roughly calculating the potential events in each standardized battery recycling process. The event values for each process from prior studies were used to derive probability factors for each process.

### 2.2. Risk Assessment Code

For process hazard analysis, this study used the Risk Assessment Code (RAC) as described in the U.S. Department of Defense (DOD) “Standard Practice System Safety” guidelines, specifically MIL-STD-882E [19]. The RAC calculates risk ratings based on severity and frequency. Table 2 categorizes severity into four levels: Catastrophic, Critical, Marginal, and Negligible. Table 3 categorizes frequency into six levels: Frequent, Probable, Occasional, Remote, Improbable, and Eliminated. Table 4 presents a Risk Assessment Matrix combining Table 2 and Table 3. Table 2, Table 3 and Table 4 show the severity categories, probability levels, and risk assessment matrix, respectively, which were adapted from MIL-STD-882E (U.S. Department of Defense, 2012).

In this study, the risk assessment focused on the severity of chemicals used in the hydrometallurgical process during the post-treatment stage, particularly in the chemical extraction steps for recovering metals such as manganese, cobalt, and nickel. The probability of accidents was assessed by combining the accident frequencies from prior studies with the identified events in the generalized battery recycling process, leading to the derivation of a probability factor for risk evaluation.

### 2.3. Severity

For the risk assessment using the RAC, the severity of chemicals was categorized using the NFPA fire diamond system. Severity was determined by referencing the classification system outlined in the National Fire Protection Association’s (NFPA) NFPA 704: Standard System for the Identification of the Hazards of Materials for Emergency Response. This standard, commonly known as the NFPA fire diamond, provides a method for hazard assessment within the battery recycling process [20].

The NFPA fire diamond, also known as NFPA 704, is a hazard rating standard established by the NFPA in the United States. Table 5 is a detailed description of each NFPA rating. This system divides hazards into four main categories and assigns a rating to each. The four-symbol system generally provides information on Health (the degree of harm to health), Flammability Hazard, Instability (chemical reactivity), and Special Hazards. Each category is rated on a scale from 0 to 4, with 0 indicating “No Hazard” and 4 indicating “Extreme Hazard”.

### 2.4. Probability

The probability of accidents occurring during the battery recycling process was calculated by combining the analysis of prior research with the events identified in this study to derive a probability factor. Specifically, the 2021 WEEE (Waste Electrical and Electronic Equipment) report, which included a survey of workers in the battery recycling industry, provided data on the proportion of incidents involving thermal events reported in 2018 [21]. The WEEE report categorized the processes into six stages: collection of WEEE, sorting of WEEE, pretreatment of WEEE (dismantling, depolluting), shredding (e.g., crushing, pressing, cutting), post-shredding treatment, and other treatment. The percentage of those declaring a thermal event was used as a reference for the probability factor in the battery recycling process. Subsequently, the probability factor for each stage of the battery recycling process was calculated by multiplying the reported incident rate with the event ratios identified in the generalized battery recycling process from prior research.

## 3. Results 1: Standardization of the Battery Recycling Process

### 3.1. Analysis of Battery Recycling Process

#### 3.1.1. Pretreatment Process

The battery pretreatment process involves seven stages: classification, stabilization, discharge and deactivation, dismantling, thermal treatment, shredding, and sorting. The classification process sorts batteries based on appearance, electrical parameters, machine learning models, and chemicals [22]. The stabilization stage includes discharging and thermal deactivation processes. Battery discharging and deactivation remove the explosion risk by discharging the battery. Discharging is primarily carried out using “wet discharge”, which uses saline solutions, or “dry discharge”, which employs electrical devices [23]. Wet discharge involves immersing the battery in a saline electrolyte to naturally induce a current and discharge the battery. Wet discharge can rapidly discharge large quantities of batteries simultaneously but may cause environmental pollution due to the wastewater generated during the process [24]. Dry discharge, efficient in discharging, avoids contamination from saline solutions affecting the battery pack’s cables and cases. It results in higher resource recovery rates and lower risks of fire and explosions, but it faces challenges in large-scale and high-speed discharging [25].

The dismantling process mechanically disassembles discharged battery packs into modules or cells [26]. Following dismantling, thermal treatment involves placing the discharged cells in organic solvents and heating them at 100–200 °C to improve recycling yields. This process separates the battery’s anode/cathode active materials and anode/cathode metal electrodes by removing binders and electrolytes [27]. Sometimes, specific stages of the pre-processing may be omitted, or one stage or another may be chosen to achieve specific purposes. For example, either solvent dissolution or thermal treatment, or both, can be adopted to remove the polyvinylidene fluoride (PVDF) binder [28]. Shredding then crushes the spent batteries into a mixed powder called black mass, containing metals like lithium and nickel. Shredding methods include wet and dry shredding. Wet shredding can reduce the particle size of the black mass but may dissolve fine active electrode materials in water, causing additional losses. Consequently, dry shredding, which enhances efficiency and maximizes selective shredding, is predominantly used [29]. Recently, crushing has mainly been carried out under inert conditions or at cryogenic temperatures [30]. When the crushing process is carried out with cryogenic liquid nitrogen, the reactivity of lithium is reduced to 1/250,000 [31]. In addition, crushing is performed in an inert atmosphere using inert gases such as Ar, N_2_, or CO_2_ to prevent spontaneous ignition or the formation of toxic gas in advance [32].

The sorting process classifies materials such as separators, cathodes, anodes, and external cans based on various physical properties such as particle size, density, weight, and magnetic characteristics. This stage separates metals like iron, copper, and aluminum [33].

#### 3.1.2. Post-Treatment Process

Post-treatment processes are generally divided into pyrometallurgy and hydrometallurgy. The pyrometallurgy does not require pretreatment and involves melting discharged batteries at high temperatures in a furnace to create alloys. This is followed by smelting to recover metals like copper, iron, nickel, and cobalt [34]. The pyrometallurgy is carried out in two stages. In the first stage, organic solvents and electrolytes are removed at temperatures between 150 and 500 °C. In the second stage, high temperatures above 1000 °C and reducing agents are used to separate the material into a lower alloy layer and an upper slag layer based on melting points and density differences. Metals such as copper, cobalt, and nickel are recovered from the lower alloy layer, while aluminum, nickel, and manganese are recovered from the upper slag layer using wet metallurgy processes [35]. The pyrometallurgy does not require the dismantling of batteries from packs to modules and cells, allowing for the recycling of large quantities of batteries. However, the process requires reprocessing to recover metals, leading to higher costs [36].

The hydrometallurgy involves recovering metals from black mass obtained through pretreatment or from slag and dust generated by the pyrometallurgy using chemical treatments with inorganic and organic solvents, acids, or bases. The hydrometallurgy includes leaching and extraction and recovery stages. Leaching involves alkaline treatment with sodium hydroxide, organic acid treatment with acetic and citric acids, or inorganic acid treatment with sulfuric and nitric acids. These methods induce metal oxidation to recover metals in ionized forms [36]. The recovery efficiency varies depending on the leaching method used. The extraction and recovery stage crystallizes metals from the leachate, separating and recovering them with high purity. The hydrometallurgy offers high metal purity and economic advantages but involves complex handling of various chemicals and generates wastewater, presenting chemical risks [37].

### 3.2. Review of Previous Studies on Battery Recycling

Research on battery recycling technologies is diverse, focusing on methods for recovering specific materials or the substances used in the recovery processes. Based on this, the research categorizes battery recycling processes by the methods used for material recovery and the substances employed.

#### 3.2.1. Retriev Technologies 

**Figure 1:**
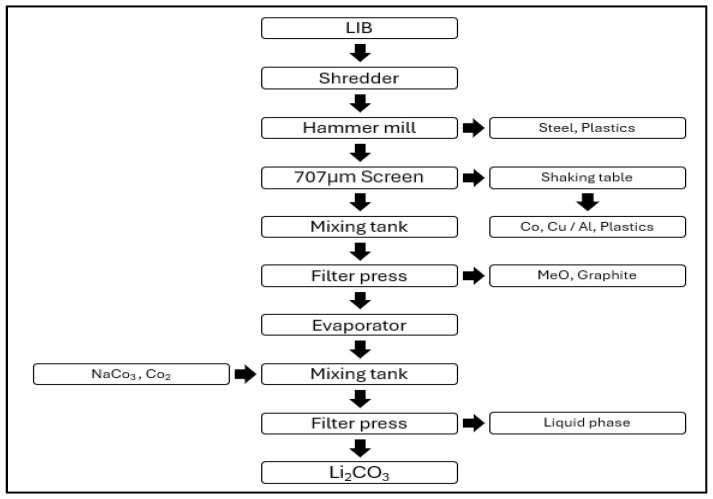
Retriev LIB recycling process.

Figure 1 shows the battery recycling process of Retriev Technologies, Inc.’s. Retriev Technologies, Inc.’s (Lancaster, OH, USA) battery recycling process consists of disassembly and discharging, shredding, separation, and cake processing stages. In the disassembly stage, large lithium-ion battery (LIB) packs are manually disassembled, while smaller batteries and cells do not undergo a disassembly process. During the discharging stage, saline discharging is employed to prevent fires that could result from lithium oxidation and to deactivate the cells [38].

In the shredding stage, discharged LIBs immersed in saline are shredded, and the resulting slurry is further ground using a hammer mill. Larger metal particles are separated by screening. In the separation stage, the slurry, primarily composed of Cu-Co after removal of aluminum particles and plastics, is filtered to produce a cake rich in carbon and metal oxides. The filtered liquid, which is rich in lithium, reacts with Na_2_CO_3_ or CO_2_ to produce Li_2_CO_3_ [38].

The chemicals used in the recycling process at Retriev Technologies, Inc. include Na_2_CO_3_ and CO_2_ for lithium extraction, and NaCl for the discharging process.

#### 3.2.2. Recupyl Valibat

**Figure 2:**
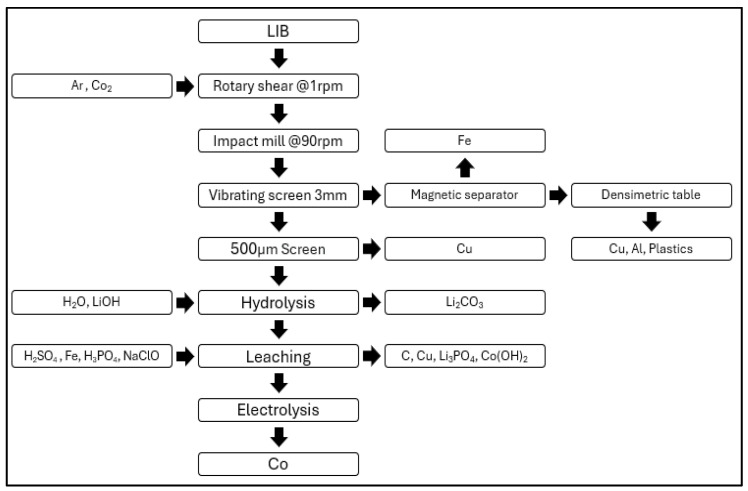
Recupyl recycling process.

Figure 2 shows the battery recycling process of Recyupyl. The Recupyl SAS Valibat battery recycling process consists of shredding, separation, liquefaction, and leaching stages. In the shredding stage, a low-speed rotary shear is used to shred the batteries, and CO_2_ is employed to reduce the fire risk associated with LIBs. During the separation stage, particle size is controlled using screens, and non-magnetic fractions are processed with a density separator to separate high-density copper and aluminum from low-density paper and plastic fractions [39]. In the liquefaction stage, fine fractions rich in electrodes are mixed with water. This process releases hydrogen, and lithium salts dissolve into the aqueous solution, forming solid metal oxides. The leaching stage involves treating the solid fractions with H_2_SO_4_, after which carbon is filtered out from the solution. Copper is cemented and reduced to its elemental form, while the remaining lithium precipitates as LiPO_4_ [39]. The chemicals used in the Recupyl SAS Valibat process include CO_2_ and H_3_PO_4_ for lithium leaching and NaClO for cobalt extraction.

#### 3.2.3. Accurec Recycling GmbH

**Figure 3:**
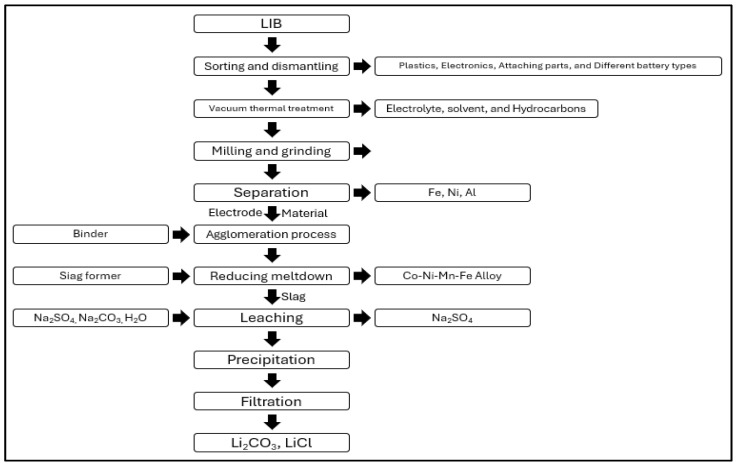
Accurec recycling process.

Figure 3 shows the battery recycling process of Accurec. The Accurec Recycling GmbH battery recycling process consists of separation, purification and disassembly, vacuum thermal treatment, shredding and grinding, mechanical separation, agglomeration and secondary thermal treatment, lithium recovery, and processing stages [40]. In the vacuum thermal treatment stage, the material is processed at a vacuum of 250 °C to remove electrolytes, solvents, and volatile hydrocarbons. During the mechanical separation stage, Fe-Ni and Al-Cu compounds are formed to extract non-metals [41]. In the lithium recovery and processing stage, H_2_SO_4_ is used to leach lithium, and Na_2_SO_4_ is obtained as a byproduct. The extracted lithium is then precipitated as Li_2_CO_3_.

#### 3.2.4. Closed Loop

**Figure 4:**
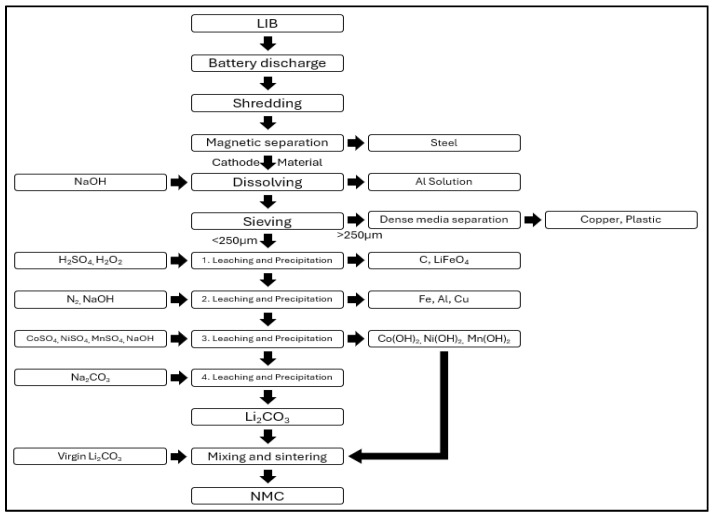
Closed loop recycling process.

Figure 4 shows the battery recycling process of Closed Loop. The “Closed Loop” battery recycling process involves several stages. After the lithium-ion batteries (LIBs) have undergone discharge, they are crushed and then separated into ferromagnetic and non-ferromagnetic materials. The ferromagnetic materials are mixed with NaOH to extract aluminum. Coarse materials are separated through density separation to recover copper, while fine particles are transferred to a resin extraction stage. The resin extraction process consists of three stages. In the first stage, carbon, LiFePO_4_, and plastics are removed. The second stage produces a solution containing Co, Ni, Mn, Li, Al, and Cu ions. In the third stage, MnSO_4_, NiSO_4_, and CoSO_4_ are added to obtain Co, Mn, and Ni in a 1:1:1 ratio [42]. Subsequently, NaOH is added to increase the pH of the solution, followed by the addition of Na_2_CO_3_ to precipitate Li_2_CO_3_ [43]. The previously extracted Co(OH)_2_, Mn(OH)_2_, and Ni(OH)_2_ are then mixed with the precipitated Li_2_CO_3_. This mixture is compressed into pellets and sintered at 900 °C to synthesize the cathode materials. The chemicals used in the “Closed Loop” battery recycling process include MnSO_4_, NiSO_4_, and CoSO_4_ for precipitating Mn, Ni, and Co, and Na_2_CO_3_ for the extraction of lithium.

### 3.3. Detailed Chemical Analysis of Hydrometallurgical Process Stages

**Figure 5:**
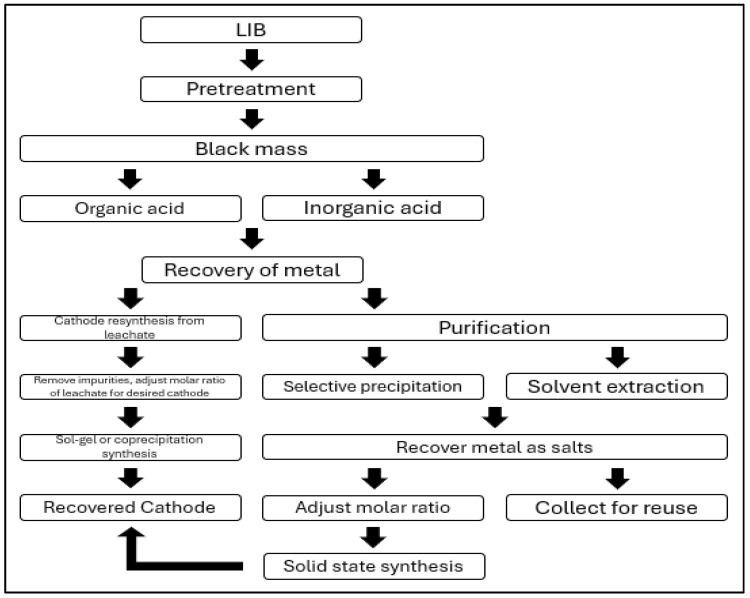
Traditional recycling process.

Most battery recycling processes primarily utilize hydrometallurgical methods. While these methods are simpler compared to pyrometallurgical processes, they exhibit lower energy efficiency and can lead to secondary pollution during battery recycling [44]. Therefore, this study focuses on assessing the risks associated with the chemicals used in hydrometallurgical processes, which dominate battery recycling. A schematic diagram of the hydrometallurgical process is shown in Figure 5 below [45].

The process of recycling spent lithium-ion batteries through hydrometallurgical methods involves obtaining black powder through pretreatment, followed by reductive acid leaching. After solid–liquid separation, the recovery of metal salts or complex salts is achieved through further separation and purification, and the materials are regenerated into cathode materials.

#### 3.3.1. Hydrometallurgical Process—Leaching Process

The leaching process involves recovering metals such as nickel and cobalt from black powder, which is a cathode active material obtained from the pretreatment process [34]. During leaching, various chemicals are used with the black powder, including alkaline substances, organic acids, and inorganic acids. Currently, among inorganic acids such as sulfuric acid, hydrochloric acid, and nitric acid, sulfuric acid is predominantly used, with hydrogen peroxide added as a reductant to facilitate metal leaching [30]. Organic acids, such as lactic acid, can also be used, though they are associated with high costs [45,46]. Common alkaline substances include ammonia, ammonium carbonate, ammonium sulfate, and ammonium chloride [47]. 

#### 3.3.2. Hydrometallurgical Process—Separation and Recovery Process

Following the leaching process, metals are separated using methods such as precipitation, solvent extraction, ion exchange, and displacement. Chemicals used for precipitation include NaOH, C_2_H_2_O_4_, H_3_PO_4_, Na_2_CO_3_, and Na_2_S [48,49,50,51]. Solvent extraction involves using a two-phase system of organic and aqueous phases to distribute and separate different metal ions. Commonly used extractants in this method are Cyanex 272, D_2_EHPA, PC88A, and Versatic acid 10 [52]. After solvent extraction, a washing process is conducted to remove co-extracted impurities, and a stripping process is used to remove impurities from the organic phase.

##### Hydrometallurgical Process—Single Component Extraction Process

The single component extraction process involves extracting individual metals such as Ni, Co, Mn, and Li from batteries after pretreatment, using a hydrometallurgical process for post-treatment. In this process, various chemicals are employed to extract specific single components.

**Figure 6:**
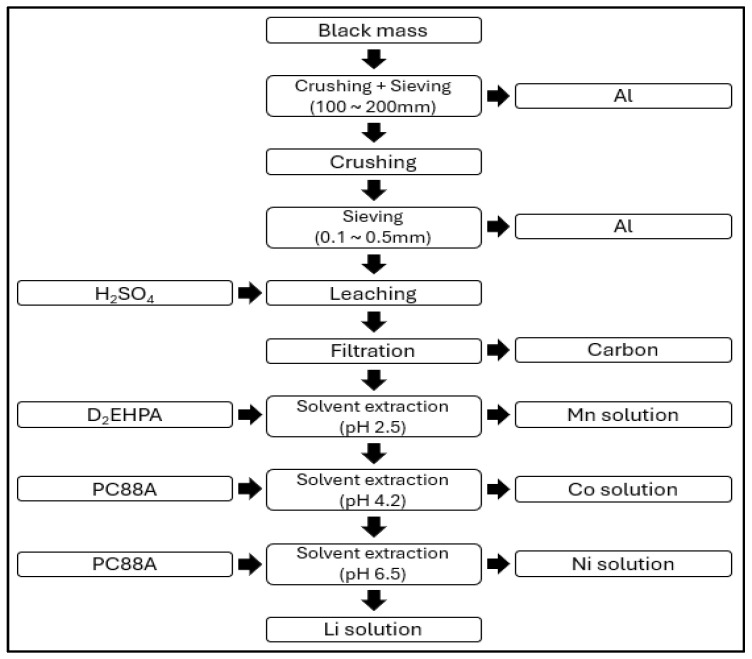
Japan’s JX Nippon battery recycling process.

Figure 6 is the process developed by JX Nippon that involves several stages. After pretreatment, the black mass is subjected to crushing and screening to remove impurities such as aluminum. The leaching process is then carried out using sulfuric acid (H_2_SO_4_). For the extraction of manganese (Mn), D_2_EHPA is utilized, while PC88A is used for the extraction of cobalt (Co). For the separation of nickel (Ni) and lithium (Li), PC88A is also employed [53].

**Figure 7:**
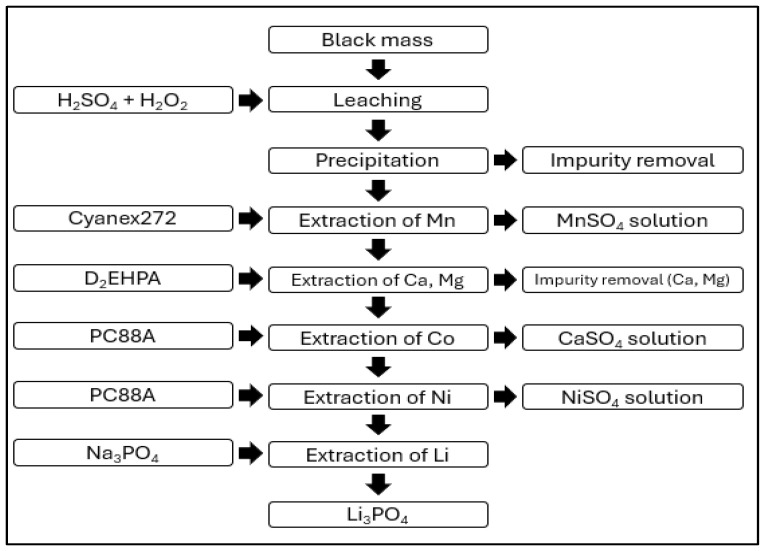
China’s Huayou Cobalt battery recycling process.

Figure 7 is the process developed by Huayou Cobalt that involves pretreating the black mass, which is then subjected to a leaching process using sulfuric acid (H_2_SO_4_) and hydrogen peroxide (H_2_O_2_). After leaching, impurities are removed through precipitation. For the extraction of manganese (Mn), Cyanex272 is used, while calcium (Ca) and magnesium (Mg) impurities are removed using D_2_EHPA. Cobalt (Co) and nickel (Ni) are extracted using PC88A. Lithium (Li) is subsequently extracted using sodium phosphate (Na_3_PO_4_) [54].

**Figure 8:**
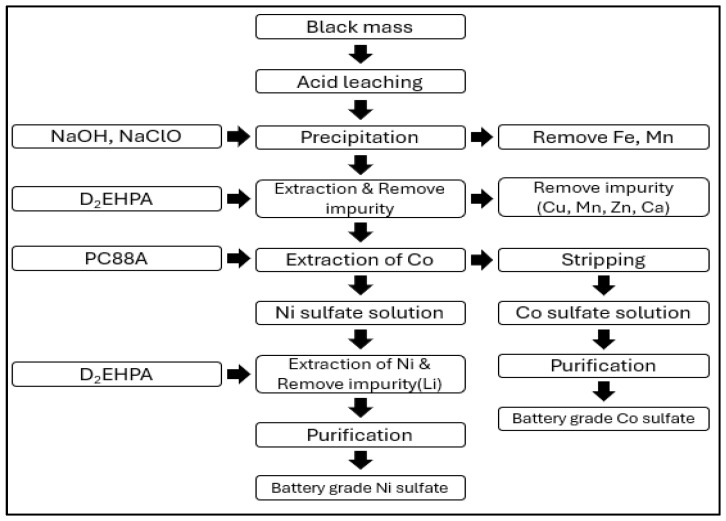
GaNPower battery recycling process.

As follows in Figure 8. GaNPower International, Inc. employs a process where the black mass, obtained after pretreatment, undergoes leaching with acids followed by a precipitation process to remove impurities such as iron (Fe) and manganese (Mn). Remaining impurities are then removed using D_2_EHPA. For the extraction of cobalt (Co), PC88A is used, while nickel (Ni) is extracted using D_2_EHPA [54].

**Figure 9:**
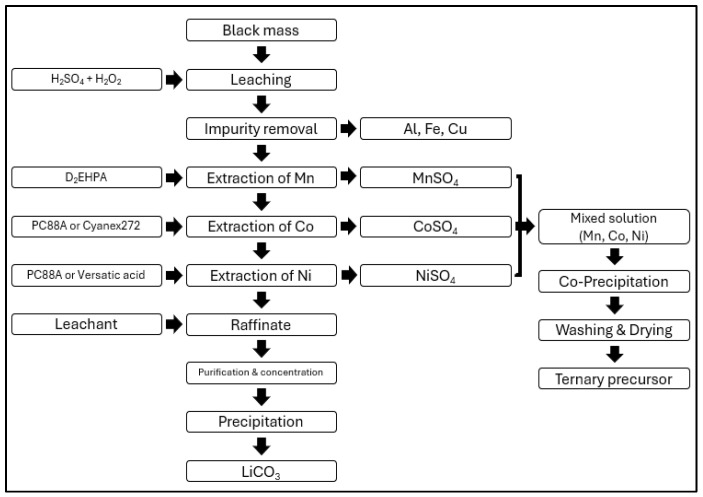
Traditional single-component battery recycling extraction process.

Figure 9 illustrates the schematic of single-component battery recycling extraction processes, with a focus on the substances used for extracting manganese (Mn), cobalt (Co), nickel (Ni), and lithium (Li) in hydrometallurgical processes. Typically, sulfuric acid (H_2_SO_4_) and hydrogen peroxide (H_2_O_2_) are used in the leaching process. For Mn extraction, D_2_EHPA is commonly employed, while Co is extracted using PC88A and Cyanex272. Nickel is extracted using PC88A and Versatic acid 10 [52,53].

### 3.4. Generalization of Processes

**Figure 10:**
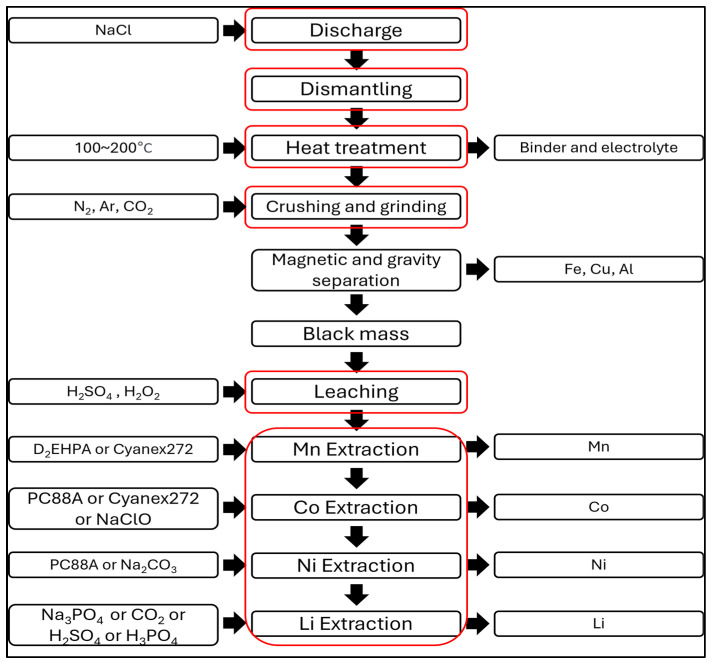
Standard battery recycling process.

To conduct a risk assessment of battery recycling processes, the recycling process has been generalized. The pre-processing phase has been standardized to include classification, wet discharging, dismantling, thermal treatment, crushing, and magnetic and gravity separation processes. Wet discharging was selected during the pre-processing phase due to its high-risk nature associated with the use of chemicals during the discharging process. For the post-processing phase, the generalization focuses on the leaching and extraction processes of the black mass created after the pre-processing crushing stage. The types, quantities, and temperature conditions of the chemicals used in the leaching and extraction processes vary according to the recycling objectives. Therefore, the generalization focuses on the commonly used hydrometallurgy in the recycling field. The extraction process is predominantly conducted using hydrometallurgical methods due to their high recovery rates for metals like Co, Ni, Mn, and Li, the use of various chemicals, and their ability to handle large volumes of batteries. The representative extraction methods include chemical precipitation, solvent extraction, ion exchange, and substitution techniques. Among these, solvent extraction was chosen for generalization due to its ability to distinguish between different chemicals used for extracting specific substances. Solvent extraction involves separating substances into two phases: aqueous and organic [54]. Different chemicals are used for each substance to be extracted in solvent extraction. The generalized processes described above are summarized in the schematic diagram shown in Figure 10, and the chemicals used in battery recycling processes are compiled in Table 6. The frames shown in red in Figure 10 represents the 6 stages of the standardized battery recycling process.

## 4. Result 2: Risk Estimation: Focus on the Hydrometallurgical Process

### 4.1. Severity

Table 7 explains the criteria for categorizing severity based on the NFPA diamond standard. According to the NFPA diamond criteria, if the sum of health, fire, and reactivity is 5~6, it is classified as Catastrophic; 3~4 as Critical; 1~2 as Marginal; and less than 1 as Negligible. In NFPA diamond, blue represents health, red represents fire, and yellow represents reactivity. Table 8 shows the severity of substances used in each process based on the NFPA diamond standard. After selecting the substances used during the discharge, crushing and grinding, leaching, and Mn, Co, Ni, and Li extraction processes in battery recycling, the severity for each substance was derived. Among all substances used in the processes, the one with the highest severity is H_2_O_2_, used in the leaching process, with a health rating of 3, fire rating of 0, and reactivity rating of 3. The second highest in severity is H_2_SO_4_, used in both Li extraction and leaching processes, with a health rating of 3, fire rating of 0, and reactivity rating of 2. The substances with the lowest severity are NaCl used in the discharge process and Ar used in the crushing and grinding processes, which all have zero health, fire, and reactivity ratings.

### 4.2. Probability

To conduct a risk assessment in the battery recycling process, we broadly identified various accident possibilities including mechanical, human, physical, and chemical factors across the six generalized stages of the process that we had previously distinguished. The main events identified are fire, electric shock, leakage, explosion, and crushing, which are the most frequently occurring events in battery recycling processes. Table 9, Table 10, Table 11, Table 12, Table 13 and Table 14 explains these events and their reasons for occurrence.

The events for each process were estimated as follows: six events for discharge, four events for dismantling, four events for thermal treatment, seven events for crushing, five events for leaching, and six events for extraction. In the extraction process, different chemicals are used for the extraction of Mn, Co, Ni, and Li, so the events were determined assuming only one chemical is used at a time. As a result, a total of 32 events were identified across all processes. The probability of each event relative to the total events was then calculated, with the results summarized in Table 15 below.

According to the WEEE report, the battery recycling process is categorized into six stages: pretreatment of WEEE (including dismantling and depolluting), shredding (e.g., crushing, pressing, cutting), and post-shredding treatment. These stages were generalized for battery recycling processes, and the distribution of these stages is detailed in Table 16.

The probability factor values for the generalized battery recycling processes were calculated by multiplying the percentage of thermal events reported with the event probability for each stage. These values were then classified according to the RAC matrix for risk assessment as outlined in Table 17. The resulting probability factor values are summarized in Table 18. In this study, the event probability was developed to represent the relative proportion of major safety incidents that could occur at each stage of the battery recycling process. This probability is intended to provide an estimation of how likely it is for safety incidents to occur within each process stage, based on the identified major events. However, it is important to note that these values are relative proportions rather than absolute, quantitative values. As such, they serve as an internal metric to compare risks across different process stages but are not directly suitable for use as quantitative measures of risk. To incorporate a more standardized and quantitative basis into our risk assessment, we utilized frequency values from the WEEE report. The WEEE report provides established frequency data regarding thermal events in battery recycling, allowing us to reference quantitative values that reflect actual incident rates. By multiplying these quantitative frequency values with the relative event probabilities identified for each stage in our study, we were able to derive the final probability factor. This probability factor thus combines the event probability as a stage-specific measure of relative likelihood with the WEEE report’s frequency values as a quantitative foundation. In summary, this approach enabled us to translate the qualitative and relative event probabilities into a more comprehensive and quantitatively supported risk estimation for each process stage in battery recycling. The probability factor therefore reflects both the unique risks identified within each stage and the standardized incident frequencies observed in the WEEE report, leading to a more robust and holistic risk assessment.

### 4.3. RAC Matrix

Table 19 summarizes the severity and probability of materials used for each battery recycling process. Table 20 is expressed in the RAC matrix. It can be seen that the processes of discharge, decomposition, and heat treatment, which are the pretreatment processes of battery recycling, are relatively safe. However, the risk was high because more relatively dangerous chemicals were used than the pretreatment processes in the leaching and extraction processes, which are not used in any post-treatment process. In particular, the use of H_2_SO_4_in the lithium extraction process has the greatest risk. It has the smallest risk in the heat treatment and decomposition processes without using chemicals during the process. It was confirmed that the processes entering with high risk based on the RAC matrix are D, F, G, H, I, J, K, L, M, N, O, Q, R, and S, the processes with serial risk are P and E, and the medium processes that are low risk are A, B, and C.

## 5. Conclusions

In this study, we generalized the stages of battery recycling processes for academic analysis through a review of prior research and the literature. We performed a relative risk assessment focusing on the substances used in each stage of the battery recycling process. The process generalization, based on various literature sources, led to the selection of hydrometallurgical processes, which use a large amount of chemicals, as the target for risk analysis. Within hydrometallurgical processes, we further generalized the process of extracting single substances. Consequently, the battery recycling hydrometallurgical process was categorized into six stages: discharge, dismantling, heat treatment, crushing, leaching, and extraction. We also collected information on chemicals used in the recycling process from the previous literature. It was confirmed that NaCl is used during discharge, CO_2_, Ar, and N_2_ during crushing and grinding, H_2_SO_4_ and H_2_O_2_ during leaching, D_2_EHPA and Cyanex 272 during Mn extraction, NaClO, PC88A, and Cyanex 272 during Co extraction, PC88A and Na_2_CO_3_ during Ni extraction, and Na_3_PO_4_, CO_2_, H_2_SO_4_, and H_3_PO_4_ during Li extraction.

To assess risk using the RAC matrix, we derived the severity and probability. Severity was determined using the NFPA 704 regulations, specifically the NFPA diamond, with H_2_O_2_ identified as the substance with the highest severity. Probability was assessed by identifying potential events in the six generalized process stages and deriving probabilities for each process. The crushing and grinding process showed the highest probability. By combining the derived probability and severity, we constructed the RAC matrix, revealing that the processes with the lowest risk are dismantling and heat treatment, while the process with the highest risk is the use of H_2_SO_4_ in Li extraction.

The findings of this study highlight that the most hazardous process in battery recycling is the use of H_2_SO_4_ during Li extraction. However, since this study is a qualitative risk assessment that does not account for the quantities of chemicals used, further research is needed to address this limitation. The quantity of substances can affect both severity and probability. Future research should also include comparative studies with actual process data from battery recycling companies. This approach will enhance the practical utility of the research for industry rather than just for academic purposes. Additionally, it is recommended that future studies use the probability factors derived from this research as a basis for quantitative risk assessment in battery recycling processes

## Figures and Tables

**Table 1:** Summary of previous studies.

Title	Author	Summary of the Paper	Ref.
Recycling end-of-life electric vehicle lithium-ion batteries	Meng-yuan Chen et al.	The research on dry smelting, wet smelting, and direct recycling methods of LIB recycling and the status of industrial development are summarized through literature and empirical examples.	[10]
Recycling of spent lithium-ion batteries in view of lithium recovery: a critical review	Chunwei Liu et al.	Among the recent technologies of LIB recycling technology, research was conducted with an emphasis on lithium recovery	[11]
Recycling of batteries: a review of current processes and technologies	A.M Bernardes et al.	Presents the current state of technology related to the collection, sorting, and processing of portable batteries	[12]
A brief review on hydrometallurgical technologies for recycling spent lithium-ion batteries	Chagens, A. et al.	Recent leaching and solvent extraction strategies for recovering valuable metals from waste lithium-ion batteries are reviewed and technological advances are discussed	[13]
Hydrometallurgical separation of aluminium, cobalt, copper, and lithium from spent Li-ion batteries	Daniel Alvarenga Ferreira et al.	A wet smelting path based on a leaching–crystallization step to separate metal Al, Co, Cu, and Li from lithium-ion batteries was evaluated	[14]
A review of physical processes used in the safe recycling of lithium-ion batteries	Roberto Sommerville et al.	The study focused on the commonly used physical processes used before the chemical treatment and purification steps during the lithium-ion battery recycling process	[15]
Recovery of cobalt sulfate from spent lithium-ion batteries by reductive leaching and solvent extraction with Cyanex 272	Jingu Kang et al.	Using Cyanex 272, we explained the chemical reaction for cobalt sulfate extraction and derived the result of extracting 92% cobalt	[16]
Comparison of two acidic leaching processes for selecting the effective recycle process of spent lithium-ion battery	Jeong-Soo Sohn et al.	A comparative study on the acid leaching process using hydrogen peroxide and oxalic acid during waste lithium-ion battery recycling process was conducted	[17]
Lithium-ion batteries as ignition sources in waste treatment processes: a semi-quantitate risk analysis and assessment of battery-caused waste fire	Thomas Nigl et al.	The risk of waste management systems was assessed based on frequency and severity by identifying the major risk factors for waste lithium-based batteries	[18]

**Table 2:** Severity categories.

Severity Categories		
Description	Severity Category	Mishap Result Criteria
Catastrophic	1	Could result in one or more of the following: death, permanent total disability, irreversible significant environmental impact, or monetary loss equal to or exceeding USD 10M.
Critical	2	Could result in one or more of the following: permanent partial disability, injuries or occupational illness that may result in hospitalization of at least three personnel, reversible significant environmental impact, or monetary loss equal to or exceeding USD 1M but less than USD 10M.
Marginal	3	Could result in one or more of the following: injury or occupational illness resulting in one or more lost workday(s), reversible moderate environmental impact, or monetary loss equal to or exceeding USD 100K but less than USD 1M.
Negligible	4	Could result in one or more of the following: injury or occupational illness not resulting in a lost workday, minimal environmental impact, or monetary loss less than USD 100K

**Table 3:** Probability levels.

Probability Levels			
Description	Level	Specific Individual Item	Fleet or Inventory
Frequent	A	Likely to occur often in the life of an item	Continuously experienced
Probable	B	Likely to occur often in the life of an item	Will occur frequently
Occasional	C	Likely to occur sometime in the life of an item	Will occur several times
Remote	D	Unlikely, but possible to occur in the life of an item	Unlikely, but can reasonably be expected to occur
Improbable	E	Incapable of occurrence. This level is used when potential hazards are identified and later eliminated	Unlikely to occur, but possible
Eliminated	F	Incapable of occurrence. This level is used when potential hazards are identified and later eliminated	Incapable of occurrence. This level is used when potential hazards are identified and later eliminated

**Table 4:** Risk assessment matrix.

Risk Assessment Matrix					
	Severity	Catastrophic (1)	Critical (2)	Marginal (3)	Negligible (4)
Probability					
Frequent (A)		High	High	Serious	Medium
Probable (B)		High	High	Serious	Medium
Occasional (C)		High	Serious	Medium	Low
Remote (D)		Serious	Medium	Medium	Low
Improbable (E)		Medium	Medium	Medium	Low
Eliminated (F)		Eliminated			

**Table 5:** Description by NFPA rating.

Risk Rating	4	3	2	1	0
Flammability	Will rapidly or completely vaporize at normal atmospheric pressure and temperature or is readily dispersed in air and will burn readily. Includes pyrophoric substances. Flash point below room temperature at 22.8 °C	Liquids and solids (including finely divided suspended solids) that can be ignited under almost all ambient temperature conditions. Liquids having a flash point below 22.8 °C and having a boiling point at or above 37.8 °C or having a flash point between 22.8 and 37.8 °C	Must be moderately heated or exposed to relatively high ambient temperature before ignition can occur. Flash point between 37.8 and 93.3 °C	Materials that require considerable preheating, under all ambient temperature conditions, before ignition and combustion can occur. Includes some finely divided suspended solids that do not require heating before ignition can occur. Flash point at or above 93.3 °C	Materials that will not burn under typical fire conditions, including intrinsically noncombustible materials such as concrete, stone, and sand. Materials that will not burn in air unless exposed to a temperature of 820 °C for more than 5 min.
Health	Very short exposure could cause death or major residual injury	Short exposure could cause serious temporary or moderate residual injury	Intense or continued but not chronic exposure could cause temporary incapacitation or possible residual injury	Exposure would cause irritation with only minor residual injury	Poses no health hazard, requires no precautions, and would offer no hazard beyond that of ordinary combustible materials
Instability reactivity	Substances that can easily explode or undergo rapid decomposition reactions even under normal atmospheric conditions. These materials are highly unstable at room temperature and pressure, potentially exhibiting explosive reactions with minimal stimulation.	Capable of detonation or explosive decomposition but requires a strong initiating source, must be heated under confinement before initiation, reacts explosively with water, or will detonate if severely shocked	Undergoes violent chemical change at elevated temperatures and pressures, reacts violently with water, or may form explosive mixtures with water	Normally stable, but can become unstable at elevated temperatures and pressures	Normally stable, even under fire exposure conditions, and is not reactive with water

**Table 6:** Substances used by process.

Process	Materials
Discharge (salt water)	NaCl
Crushing and grinding	CO_2_
	Ar
	N_2_
Leaching	H_2_SO_4_
	H_2_O_2_
Mn extraction	D_2_EHPA
	Cyanex 272
Co extraction	NaClO
	PC88A
	Cyanex 272
Ni extraction	PC88A
	Na_2_CO_3_
Li extraction	CO_2_
	Na_3_PO_4_
	H_2_SO_4_
	H_3_PO_4_

**Table 7:** NFPA diamond-based severity classification.

Severity	
Catastrophic (1)	5~6
Critical (2)	3~4
Marginal (3)	1~2
Negligible (4)	<1

**Table 8:** NFPA diamond and severity of each substance used in the process.

Process	Material	NFPA Diamond				Severity
		Health	Fire	Reactivity	NFPA 704	
Discharge	NaCl	0	0	0	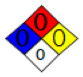	4 (0)
Crushing and grinding	CO_2_	3	0	0	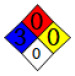	2 (3)
	Ar	0	0	0	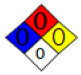	4 (0)
	N_2_	3	0	0	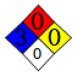	2 (3)
Leaching	H_2_SO_4_	3	0	2	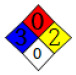	1 (5)
	H_2_O_2_	3	0	3	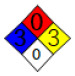	1 (6)
Mn extraction	D_2_EHPA	2	1	0	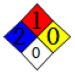	2 (3)
	Cyanex272	2	1	0	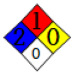	2 (3)
Co extraction	NaClO	2	0	1	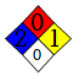	2 (3)
	PC88A	3	1	0	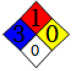	2 (4)
	Cyanex 272	2	1	0	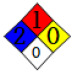	2 (3)
Ni extraction	PC88A	3	1	0	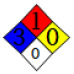	2 (4)
	Na_2_CO_3_	3	0	0	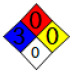	2 (3)
Li extraction	CO_2_	3	0	0	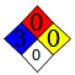	2 (3)
	Na_3_PO_4_	2	0	0	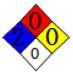	3 (2)
	H_2_SO_4_	3	0	2	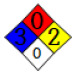	1 (5)
	H_3_PO_4_	3	0	0	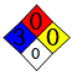	2 (3)

**Table 9:** Events in the process of discharge.

Discharge		
Result	Event	Event Reason
Accident during discharge process	Fire	A fire caused by a rise in saltwater temperature
		A fire caused by an external shock
		Fire due to short circuit during battery mass discharge
	Electric shock	Electric shock due to improper discharge time
		Electric shock due to not wearing protective gear during discharge
	Leak	Leakage of internal toxic subs

**Table 10:** Events in the process of dismantling.

Dismantling		
Result	Event	Event Reason
Accident during dismantling process	Crush injuries	Not wearing protective equipment
		Crush injuries due to lack of expertise
	Electric shock	Electric shock during disassembly of the battery that has not been discharged
	Explosion	An explosion caused by abnormal dismantling

**Table 11:** Events in the process of heat treatment.

**Heat treatment**		
**Result**	**Event**	**Event Reason**
Accident during heat treatment process	Leak	Toxic gas leakage during process
	Fire	A fire caused by a chemical reaction error
		Fault in the cooling system due to coolant spills and shortages
		Fire due to control system malfunction

**Table 12:** Events in the process of crushing and grinding.

Crushing and Grinding		
Result	Event	Event Reason
Accident during crushing and grinding process	Leak	Toxic gas leakage due to the reaction of compounds inside the battery during grinding
		A leak caused by crushing
	Fire	A fire caused by a battery failure
		Fire due to overheating of the shredder
		Fire due to chemical reactivity due to failure of inert atmosphere
		Fire due to compound reaction inside the battery during crushing
	Explosion	Explosion due to scattering dust during crushing and grinding

**Table 13:** Events in the process of leaching.

Leaching		
Result	Event	Event Reason
Accident during leaching process	Fire	Fire due to mix ratio error
		Steam vent failure in reactor
		Carry out firework in areas where flammable gases remain
	Leak	Leakage due to failure of control such as temperature, concentration, reaction time, etc.
		Leakage due to system failure

**Table 14:** Events in the process of discharge Mn, Co, Ni, Li extraction.

Mn, Co, Ni, Li Extraction		
Result	Event	Event Reason
Accident during extraction process	Fire	Fire due to process error (mixing ratio error, abnormal reaction, etc.)
	Explosion	Explosion due to process error (mixing ratio error, abnormal reaction, etc.)
	Leak	Chemical leak during Mn extraction process
		Chemical leak during Co extraction process
		Chemical leak during Ni extraction process
		Chemical leak during Li extraction process

**Table 15:** Battery recycling process accident probability.

Process		Probability
Pre-processing	Discharge	0.1875 (6/32)
	Dismantling	0.125 (4/32)
	Heat treatment	0.125 (4/32)
	Crushing and grinding	0.2188 (7/32)
Post-processing	Leaching	0.1563 (5/32)
	Extraction	0.1875 (6/32)

**Table 16:** Battery recycling processes classification and thermal event rate by process.

Process	Event Probability	Grouped by Type of Activities on-Site	% of Those Declaring a Thermal Event
Discharge	0.1875 (6/32)	Pretreatment of WEEE	0.57
Dismantling	0.125 (4/32)		
Heat treatment	0.125 (4/32)		
Crushing and grinding	0.2188 (7/32)	Shredding	0.69
Leaching	0.1563 (5/32)	Post-shredding treatment	0.77
Extraction	0.1875 (6/32)		

**Table 17:** Probability range.

Probability	
Frequent (A)	≥0.14
Probable (B)	0.12~0.14
Occasional (C)	0.1~0.12
Remote (D)	0.1≤

**Table 18:** Probability factor for each battery recycling process.

Process	Event Probability	% of Those Declaring a Thermal Event	Probability Factor	Probability
Discharge	0.1875 (6/32)	0.57	0.11	C
Dismantling	0.125 (4/32)		0.071	D
Heat treatment	0.125 (4/32)		0.071	D
Crushing and grinding	0.2188 (7/32)	0.69	0.151	A
Leaching	0.1563 (5/32)	0.77	0.1203	B
Extraction	0.1875 (6/32)		0.144	A

**Table 19:** Battery recycling process accident probability and severity.

	Process			Material	Severity	Probability
A	Pre-processing	Discharge		NaCl	4	C
B		Dismantling		-	4	D
C		Heat treatment		-	4	D
D		Crushing and grinding		CO_2_	2	A
E				Ar	4	
F				N_2_	2	
G	Post-processing	Leaching		H_2_SO_4_	1	B
H				H_2_O_2_	1	
I		Extraction	Mn	D_2_EHPA	2	A
J				Cyanex 272	2	
K			Co	NaClO	2	
L				PC88A	2	
M				Cyanex 272	2	
N			Ni	PC88A	2	
O				Na_2_CO_3_	2	
P			Li	Na_3_PO_4_	3	
Q				CO_2_	2	
R				H_2_SO_4_	1	
S				H_3_PO_4_	2	

**Table 20:** Risk assessment matrix by battery recycling process.

Risk Assessment Matrix					
	Severity	Catastrophic (1)	Critical (2)	Marginal (3)	Negligible (4)
Probability					
Frequent (A)		R	D, F, I, J, K, L, M, N, O, Q, S	P	E
Probable (B)		G, H,			
Occasional (C)					A
Remote (D)					B, C

## Data Availability

Data are contained within the article.
